# Evaluation of Clinical Effectiveness and Subjective Satisfaction of a New Toothbrush for Postsurgical Hygiene Care: A Randomized Split-Mouth Double-Blind Clinical Trial

**DOI:** 10.1155/2015/828794

**Published:** 2015-03-16

**Authors:** Marco Montevecchi, Annalisa Moreschi, Maria Rosaria Gatto, Luigi Checchi, Vittorio Checchi

**Affiliations:** ^1^Department of Biomedical & Neuromotor Sciences, School of Dentistry, Division of Periodontology and Implantology, Alma Mater Studiorum, University of Bologna, 40100 Bologna, Italy; ^2^Department of Oral Sciences, University of Trieste, 34100 Trieste, Italy

## Abstract

The aim of this RCT was to evaluate plaque control and gingival health promotion effectiveness of a new toothbrush with extra-soft filaments in postsurgical sets. Ten consecutive patients with at least two scheduled symmetrical periodontal surgeries were selected. Following the first periodontal surgery, a test (TB1) or control (TB2) toothbrush was randomly assigned. After the second surgery, the remaining toothbrush was given. Patients were asked to gently wipe the surgical area from days 3 to 7 postoperatively and to gently brush using a roll technique from day 7 till the end of the study. Baseline evaluation took place on the day of surgery and follow-ups were performed at days 7, 14, and 30 postoperatively. A more evident PI reduction was recorded for test toothbrush where a regular decrease was observed till day 14; then, this parameter tended to stabilize, remaining however lower than that recorded for the control toothbrush. There were no statistical differences in the GI between test and control toothbrushes. All patients introduced the test toothbrush at surgical site at third day; the control toothbrush was introduced within a mean of 9 days. The introduction of the test toothbrush 3 days after periodontal surgery may be recommended.

## 1. Introduction

As clearly stated by the 1999 Second World Conference on Oral Health Promotion in a consensus report on oral hygiene, bacterial plaque plays a crucial role in dental caries and periodontal diseases aetiology [1]. Therefore, an effective removal of dental plaque can be considered essential to maintain the oral state of health.

The relevance of a thorough oral plaque control is also correlated to a favourable postsurgical wound healing [2]. Interestingly, this clinical circumstance is characterized by the inhibitory effect of bacterial contamination on wound healing [2–5]. In 1994, the First European Workshop on Periodontology declared that the maintenance of a high standard postsurgical plaque control is mandatory for a successful outcome of periodontal surgery [6].

A variety of debridement and anti-infective measures have been advocated to improve clinical outcomes following surgical intervention; however only a few studies have addressed the role of mechanical plaque control [7–10]. There is limited evidence describing optimum timing, frequency, and detailed technique of patient-performed mechanical debridement immediately after oral surgical procedure [11–15].

Nowadays, several toothbrushes dedicated to postsurgical hygiene are available but unfortunately with very poor scientific support. A common characteristic of these products is the presence of thin and soft bristles, likely because of discomfort and sensitivity at the surgical site.

Recently, toothbrushes with very innovative bristles with conical shape and extra-thin end have been introduced. According to the manufacturer, the new design allows removing plaque thoroughly yet gently while reducing the mechanical tissue damage and maintaining a good hygienic level. Clinical studies were carried out on these new toothbrushes and supported this innovation both for clinical effectiveness and patient satisfaction [16–18].

More recently, a new toothbrush with ultra-soft conical bristles has been marketed for postsurgical use.

The aim of the present randomized split-mouth double-blind clinical trial was to compare the effectiveness in postsurgical plaque control, the health gingival promotion, and subjective satisfaction of a new toothbrush with conical ultra-soft bristles compared to a standard reference toothbrush with flat end rounded filaments (ADA Council on Scientific Affairs, May 1998).

## 2. Materials and Methods

### 2.1. Subjects

From the patient pool of the Division of Periodontology and Implantology, School of Dentistry, University of Bologna, Italy, a sample of patients was recruited after having signed an informed consent. Patients enrolled in the study were all diagnosed as having chronic periodontitis, in good general state of health (American Society of Anesthesiologists physical status classification: class 1 or 2), and in need of at least two periodontal surgeries in symmetrical areas. Surgeries eligible for the study were bone resective surgeries or mucogingival techniques. A balanced number of similar surgical procedures were obtained.

Requirements for inclusion in the study sample were as follows: age older than 17 years, absence of medical conditions recognized to potentially influence healing conditions, normal manual skills, full mouth O'Leary Plaque Index ≤ 20%, and full mouth bleeding score (FMBS) < 25% at reevaluation after initial therapy. Smokers were not included in the study.

All patients received systematic periodontal therapy including motivation of the patient, instruction in oral hygiene procedures, and scaling and root planning under local anaesthesia. Following this, tissues were allowed to heal for a minimum of 30 days; then the need for periodontal surgical intervention was determined.

### 2.2. Toothbrushes

In this study two toothbrushes were used: Meridol-Perio toothbrush (TB1) and a manual ADA flat trimmed reference toothbrush (TB2).

**Figure 1:**
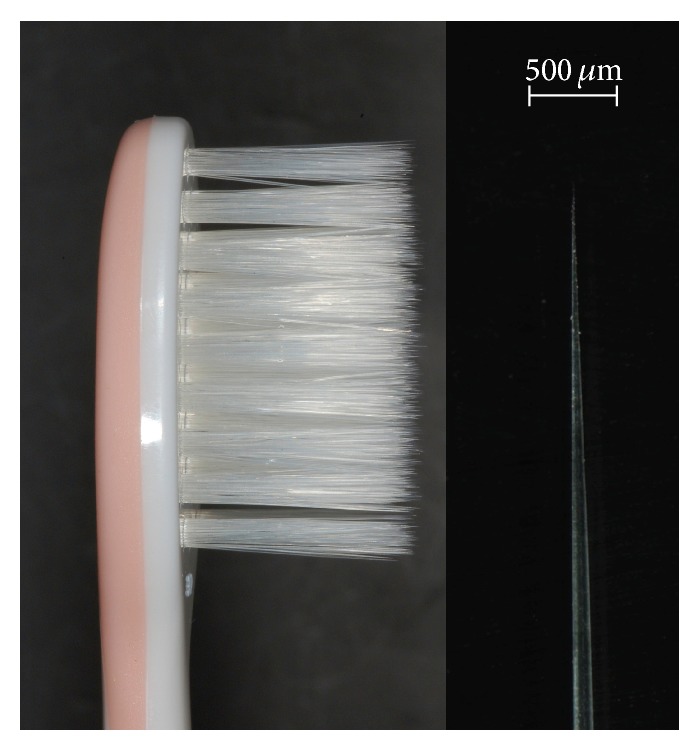
The head of the test toothbrush (TB1) and an extra-fine conical bristle.

TB1 has 37 tufts of extra-fine conical bristles arranged in 38–52 filaments per tuft. Bristles have a base diameter of 0.15 mm and 0.02 mm at the tip (Figure 1).

**Figure 2:**
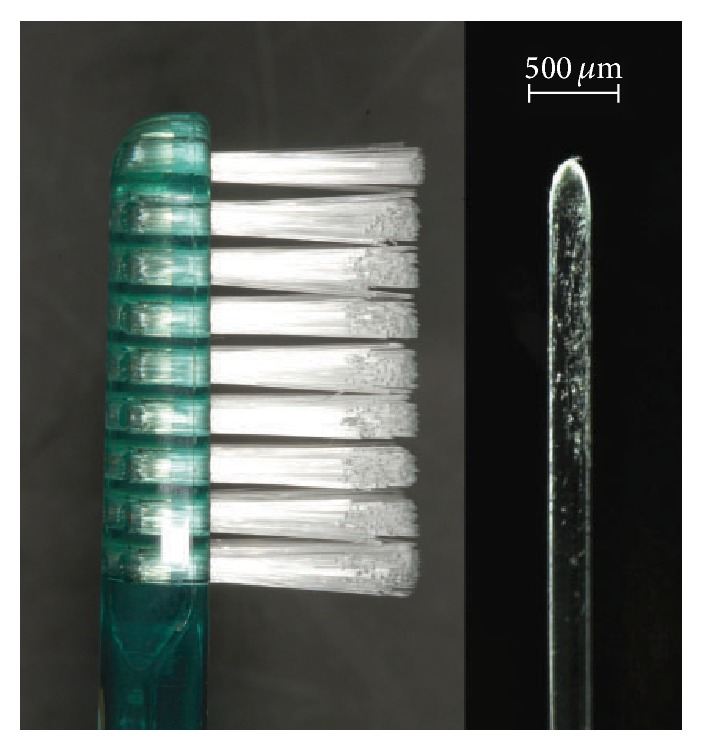
The head of the control toothbrush (TB2) and a soft-end cylindrical bristle.

TB2 has 32 tufts of soft-end cylindrical bristles arranged in 28–32 filaments per tuft (Figure 2).

### 2.3. Study Design and Experimental Procedures

Specific approval was obtained by the Scientific Board of the Faculty (Department of Odontostomatological Sciences, 26-03-2008) and participants signed informed consent forms in accordance with guidelines of the Helsinki declaration.

The study had a randomized split mouth model with two sessions whereby each subject used all brushes.

Surgical procedures were performed by the same periodontal specialist (Marco Montevecchi) and included both bone resective and mucogingival approaches. No periodontal dressing was ever applied.

Randomization of toothbrush allocation was performed using true random numbers (http://www.random.org).

Standardized perioperative analgesia consisted of 80 mg ketoprofen lysine salt sachets at the time of surgery and 4 h later, with subsequent doses as required. Sutures were removed at 7 days for resective surgery and at 14 days for mucogingival surgery.

Immediately postoperatively all patients were provided with the randomly assigned toothbrush and written oral instructions. One investigator (Annalisa Moreschi), blind from the toothbrush allocation, performed all postoperative care and instruction. The investigator also took notes of eventual adverse events at the surgical site.

Patients were instructed to brush teeth not involved in the surgical site twice daily with usual toothbrush and to clean these teeth interdentally as usual.

The patients were asked to gently wipe the surgical area with the assigned toothbrush using light vertical strokes from days 3 to 7 postoperatively and from day 7 till the end of the study to gently brush using a roll technique. The decision to restart the use of the toothbrush in the surgical area after day 3 was left to the patient depending on his/her perception of discomfort. Patients were also instructed to load toothbrush with chlorhexidine gel 0.2% (Dental Gel, Corsodyl) before wiping the dentogingival area. The toothpaste was avoided for the entire period of observation.

At each examination, teeth of the surgical sites were professionally debrided by means of a rubber cup and spongy interdental floss.

### 2.4. Assessments

Baseline evaluation took place on the day of the surgery. Follow-up examinations were performed at days 7, 14, and 30 postoperatively prior to the professional debridement. Clinical recordings were limited to the surgical area.

Plaque was assessed at each session by means of a liquid plaque disclosing agent (Red-Cote Liquid, GUM) using the O'Leary Plaque Index [19]. Six sites per tooth were scored recording presence or absence of plaque.

The Silness and Loe Gingival Index (GI) was recorded at baseline and at the 30 days of examination [20]. Before plaque disclosing, four values per each tooth were scored by means of a periodontal probe.

At the end of each session, all subjects received a questionnaire using a Visual Analogue Scale (VAS-scores) designed to evaluate the daily pain of the surgical area at toothbrush application. Subjects were asked to mark out a point on a 10 cm long calibrated line with the negative extreme response (0) on the left and the positive extreme (10) at the right end. Usage of analgesics, time, and entity were also noted.

At the last session of the second surgery, the patient was asked to express the personal preference about one of the two toothbrushes used. The document included questions about eventual limitations at use and an area for spontaneous patient's considerations.

### 2.5. Statistical Analysis

By hypothesizing that 80% of subjects prefer TB-1 with *α*-error = 0.05 and a power of 80% a sample size of 10 patients (20 surgeries) was set.

Calibration of the operator was performed through a training period of one week dedicated to measure PI on a cast covered by a known quantity of artificial plaque; as for GI ten photos with known scores of gingival status were used aiming at improving the ability of the operator in assigning the gingival mucosa to the exact score. Reliability analysis was carried out by repeating twice both PI and GI assessments at an interval of 30 minutes between the measurements on a pilot sample of ten subjects; intraclass correlation coefficient values were, respectively, equal to 0.935 (*P* = 0001) and 0.934 (*P* = 0001).

After having controlled the normality distribution of PI and GI at each time by using Shapiro-Wilk test, a Generalized Linear Model (GLM) was applied to verify the influence of the type of surgery (fixed factor) and of the toothbrush (random factor) on them, considering PI and GI at baseline as covariates.

PI trends and subjective pain were analysed by means of univariate ANOVA.

A period limited from the 3rd to the 14th day was due to the lack of pain during the following. All statistical analyses were 2-tailed; *α* level was a priori set at 0.05.

## 3. Results

A sample of 10 patients, 5 treated with osseous resective surgery and 5 with mucogingival surgery, was identified. Patients were aged between 42 and 72 years (mean 60 ± 11) with 6 females and 4 males. No dropouts during the study period occurred.

No adverse events were registered and the healing process was uneventful. Analgesic intake was always suspended before the third day after surgery.

The reintroduction of the toothbrush at surgical site was done for TB1 at third day by all patients. Conversely, TB2 was introduced within a mean of 9 days, ranging from 3 to 13 days.

The type of surgery did not influence PI at each time point; on the contrary the toothbrush type resulted in lower PI for TB1 in comparison to TB2 (Table 1).

Concerning GI after 30 days, the interaction “type of surgery” and “type of toothbrush” resulted in the limit of statistical significance (Table 2).

**Figure 3:**
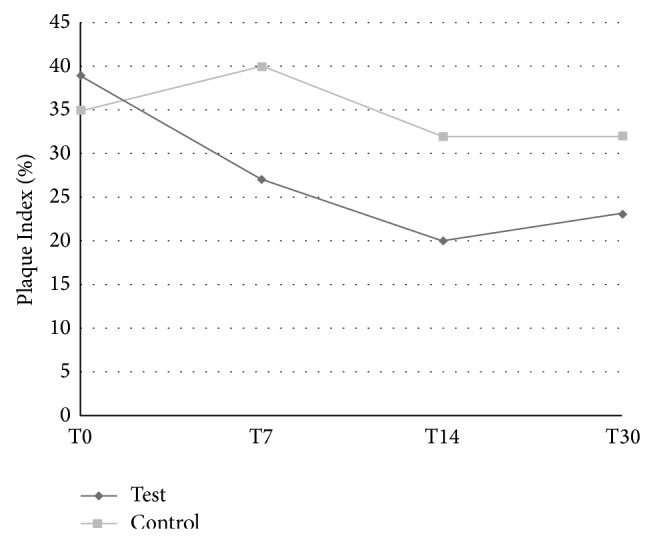
Plaque Index at each time point of the study for test (TB1) and control (TB2) toothbrushes.

A more evident PI reduction was recorded for TB1, where a regular decrease was observed till day 14; this parameter tended then to stabilize but remaining lower than that recorded for TB2 (Figure 3). The differences in PI at days 7, 14, and 30 between the toothbrushes were all statistically significant (resp., *P* = 0.002, *P* = 0.001, and *P* = 0.015).

**Figure 4:**
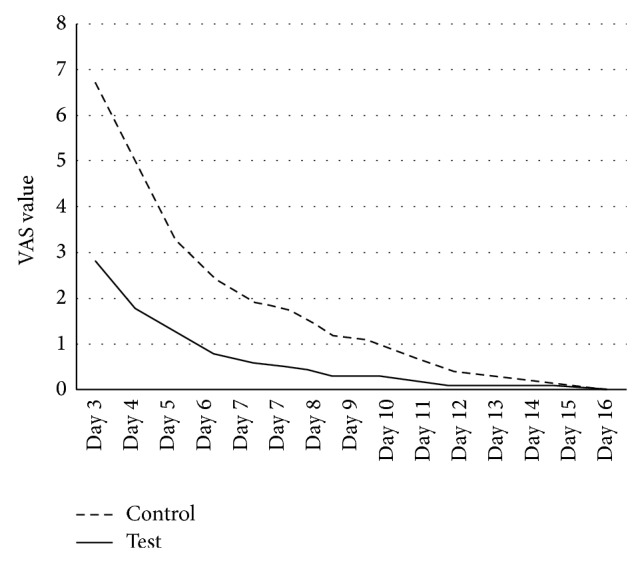
Discomfort at brushing (VAS scales: 0 = minimum, 10 = maximum) during the study period for test (TB1) and control (TB2) toothbrushes.

For both toothbrushes, the pain of brushing gradually decreased during the observation period and then disappeared at the end of the second week. The intensity of discomfort was always greater for TB2 even if not supported by a statistical significance (Figure 4).

All patients expressed a clear preference for TB1; 90% of the patients found it more comfortable to use and 56% perceived it more effective in food removal at interproximal areas. The patients reported no side effects for any of the two toothbrushes.

## 4. Discussion

The beneficial effects of optimal plaque control during the wound-healing process are clearly documented by several studies [7–9, 21, 22]. Nevertheless, a plain hygienic postsurgical protocol is still lacking.

There is a general concern about harming the wounded gingiva during the cleaning process; therefore, the general routine is to suspend the mechanical cleansing procedures for a few days or eventually use a soft toothbrush during the first days after the surgical procedure.

Limited data are anyway available supporting the widely held view that soft toothbrush bristles cause less tissue abrasion [23, 24].

Very little is also known about the early use of toothbrushes after periodontal surgery. In a RCT published in 2004 [14], Heitz et al. proposed a specific postsurgical cleansing protocol including the introduction at day 3 of mechanical cleaning by means of a very soft toothbrush. The authors recorded an optimal wound closure at 4 weeks, concluding that the use of a very soft brush did not result in any adverse event and that it may be routinely used for periodontal surgical procedures.

The present study attempts to evaluate the clinical effectiveness and the subjective satisfaction of a new extra-soft toothbrush (TB1) compared with a conventional flat trim one (TB2) over a period of 30 days after periodontal surgery. Mucogingival and osseous resective surgeries were both considered in the study. Both toothbrushes were effective in plaque removal, but TB1 showed significantly higher and faster decrees of this parameter.

The superiority of conical filaments to traditional ones in plaque removal has been clearly demonstrated in clinical and preclinical studies [17, 18, 25].

Interestingly, the reduction of PI started very early during the study for TB1 while for TB2 it started after day 7. It must be observed that only TB1 was easily introduced by each patient at day 3 and this means that plaque removal with TB1 started earlier than TB2 which was reintroduced with a mean of 9 days. It is therefore arguable that PI at the first postsurgical control was consequently lower for those sites where the toothbrush was regularly used.

In spite of that, it must be noted that at day 7 the patient was instructed to pass from gently weeping to vertical strokes. Considering this, it can be speculated that TB1 displays higher effectiveness than TB2 in plaque removal even when used in a very gentle way.

During this study no differences in terms of GI between test and control toothbrush were observed. Most likely this result can be explained by the fact that GI at day 30 reflects a generally good degree of healing. It has been previously reported that when oral disinfection is guaranteed, an optimal wound closure is present at 4 weeks following periodontal flap surgery [14]. Because softness of the surgical site was expected, during the present study measuring of GI was not carried out at each time point but only during the final analysis. At the final time point, an overall good level of healing was already present probably limiting the detection of any GI differences.

Pain at toothbrush application was generally reported till day 14. Interestingly, for those surgical areas where the TB1 was applied, pain intensity was lower and decreased faster.

The indication to introduce mechanical cleaning at day 3 was mutually taken from a previous study where the authors indicated that the first 2-3 days of wound healing are characterized by a fragile site and an inflammatory reaction to promote mechanical decontamination [14].

The absence of adverse events applying the present hygienic protocol, even when a conventional toothbrush was used, suggests the prospect of a very brief toothbrush suspension after periodontal surgery.

The subjective questionnaires revealed a clear preference for TB1 feeling it more comfortable and effective in debris removal, especially from interproximal areas.

In light of the fact that the use of the TB1 did not result in any adverse events and showed a tendency to less discomfort following surgical intervention, it may be routinely used for periodontal procedures.

Considering higher plaque reduction and faster postsurgical relief, less sensitivity, and a general preference for it, a promising potential for promoting effective plaque removal with periodontal surgery can be ascribed to TB1.

The advantages of an earlier reintroduction of the toothbrush after surgery, leading to faster clinical healing and fewer complications, are beneficial to the patient in the short term. Whether this translates to a clinically significant long-term effect is a subject for future studies.

## 5. Conclusion

The present work shows that TB1 helps in motivating patients to introduce mechanical plaque control in early postoperative period and that the use of it reduces PI to significantly low levels.

## Figures and Tables

**Table 1:** Influence of the type of surgery (fixed factor) and of the toothbrush (random factor) on Plaque Index (PI) at 7, 14, and 30 days. PI mean values and Standard Deviation (SD) at three times are reported for both test (TB1) and control toothbrushes (TB2). Generalized Linear Model applied considering PI at baseline as covariate.

	PI: 7 days	PI: 14 days	PI: 30 days
	*P* =	*P* =	*P* =
PI baseline	0.001	0.021	0.075
Surgery	0.324	0.605	0.170
Toothbrush	0.032	0.023	0.0001
Surgery and toothbrush	0.604	0.697	0.883
Test TB1 mean (SD)	27.10 (6.72)	20.10 (7.72)	23.20 (6.76)
Control TB2 mean (SD)	39.90 (8.56)	32.40 (6.15)	32.20 (8.07)

**Table 2:** Influence of the type of surgery (fixed factor) and of the toothbrush (random factor) on Gingival Index (GI) at 30 days. GI mean values and Standard Deviation (SD) are reported for both test (TB1) and control toothbrushes (TB2). Generalized Linear Model applied considering GI at baseline as covariate.

	GI: 30 days
	*P* =
GI baseline	0.002
Surgery	0.719
Toothbrush	0.299
Surgery and toothbrush	0.050
Mean (SD) TB1	0.27 (0.15)
Mean (SD) TB2	0.55 (0.29)
